# Development of vaccines at the time of COVID-19

**DOI:** 10.1093/femsml/uqaa003

**Published:** 2020-12-17

**Authors:** Jeffrey Almond, Jörg Hacker, Colin Harwood, Mariagrazia Pizza, Rino Rappuoli, Eliora Z Ron, Philippe Sansonetti, Samantha Vanderslott, Lothar H Wieler

**Affiliations:** The Sir William Dunn School of Pathology, University of Oxford, South Parks Road, Oxford OX1 3RE, UK; German National Academy of Science Leopoldina, Jägerberg 1, 06108 Halle, Germany; Centre for Bacterial Cell Biology, Biosciences Institute, Newcastle University, Baddiley-Clark Building, Newcastle upon Tyne NE2 4AX, UK; GSK Vaccines, Via Fiorentina, 1, 53100 Siena SI, Italy; GSK Vaccines, Via Fiorentina, 1, 53100 Siena SI, Italy; The Shmunis School of Biomedicine and Cancer Research, Faculty of Life Sciences, Tel Aviv University, PO Box 39040, Tel Aviv 6997801, Israel; Institut Pasteur, 25-28 Rue du Dr Roux, 75015 Paris, France; Oxford Vaccine Group and Oxford Martin School, 34 Broad St, Oxford OX1 3BD, UK; Robert Koch Institute, Nordufer 20, 13353 Berlin, Germany

**Keywords:** vaccines, COVID-19, pandemics, vaccination, vaccinology, pathogens

## Abstract

In December 2019, a working group of the European Academy of Microbiology assembled to discuss various aspects of vaccines and vaccinations. The meeting was organised by Jörg Hacker and Eliora Z. Ron and took place in the offices of the Leopoldina (German National Academy of Sciences Leopoldina). Several important issues were addressed and a major part of the discussion focused on the need to develop new vaccines, especially to protect against pathogens that constitute a pandemic threat. Following the rapid and unpredicted spread of COVID-19 in the first seven months of 2020, the need to develop vaccines for pandemic viruses rapidly has been clearly established. Thus, this paper will concentrate on points that were highlighted by the recent COVID-19 pandemic and lessons learnt therefrom.

## Teaser

Development of vaccines in the times of COVID-19.

## THE VALUE OF VACCINES

### Introduction

Infectious diseases continue to be a major factor in the development of health policy. Approximately 20% of all deaths worldwide are due to infections (Roth *et al*. 2018). One of the greatest public health achievements has been the development of preventive vaccination. Vaccines are the most cost-effective preventative measure in infectious medicine and have made a significant contribution to the containment of infectious diseases, helping to prevent up to 3 million deaths each year (UNICEF 2019). In light of their low risk and excellent cost-to-benefit ratio, vaccines have helped consolidate the concept of ‘global public health’, which is, in theory at least, applicable to any population at any latitude. The World Health Organization (WHO) was able to certify the eradication of smallpox in 1980, encouraging it and other non-governmental organisations to engage in a similar worldwide program to eradicate poliomyelitis. In addition, diphtheria, tetanus, rabies, typhoid fever, severe forms of pediatric tuberculosis, whooping cough, measles, rubella, mumps, hepatitis B and some invasive infections by encapsulated bacterial pathogens such as *Neisseria meningitidis, Haemophilus influenzae* and *Streptococcus pneumoniae* could, in principle, be added to the list of controllable if not eliminable infectious diseases. Today, however, almost all countries face problems with insufficient vaccination coverage due to various threats, such as civil strife or the opposition in some quarters to vaccination or the under-vaccination of vulnerable groups. In 2019, WHO placed vaccination hesitancy on its top 10 list of global health threats (WHO 2020b).

While vaccines are one of the most important weapons in the arsenal for combating infectious disease, other important elements of disease prevention and control should be neither forgotten nor ignored. These include, in particular, clean water, good hygiene and specific infection control measures. In terms of individual therapy, the use of antimicrobials (antibiotics and antivirals) also needs to be mentioned. The importance of these measures is illustrated, for example, by the Black Death, which raged throughout Europe between 1347 and 1351, and was responsible for wiping out between 30 and 50% of the population. The use of whole genome sequencing to understand what made the 14th-century causative agent, *Yersinia pestis*, so virulent came to the surprising conclusion that it was virtually identical (97 single nucleotide polymorphisms) to strains in circulation today and that it causes only minor outbreaks (Bos *et al*. 2011; Callaway 2011; Feldman *et al*. 2016).

Clearly, social conditions, such as nutrition, lifestyle, access to medical facilities and sanitation, combine to limit the infection and transmission of this and other microbes. The importance of sanitation is illustrated by the work of three 19th-century pioneers: Joseph Bazalgette, who built the London sewage system, John Snow, regarded as the father of epidemiology for tracing the source of a cholera outbreak in Soho, London, and Ignaz Semmelweis, the Hungarian physician responsible for developing antiseptic procedures that are now central to infection control in healthcare and other public facilities (Snow 1856; Semmelweis 1974; Lane, Blum and Fee 2010).

## THE HISTORY OF VACCINATION

Disease prevention through vaccination began more than 200 years ago in Europe with Edward Jenner and the development of the smallpox vaccine in 1796 (Riedel 2005). However, long before the 18th century, it was common practice in China and the Middle East to infect people not suffering from smallpox by inoculating the secretion from the pustules of smallpox patients. Most of these inoculated individuals fell ill comparatively easily and were protected from smallpox for the rest of their lives. However, inevitably, some individuals died from the inoculation itself (Boylston 2012). The English country doctor Edward Jenner developed a less dangerous vaccination method (Morgan and Parker 2007). He had observed that farm workers who had already been infected with the harmless cowpox were often spared the dangerous, usually fatal human pox. He concluded that people could be protected from the disease by a targeted infection with cowpox in the form of a vaccination. In order to prove a causal connection, he carried out the following decisive experiment in 1796: he vaccinated an eight-year-old boy, who had previously been spared from smallpox, with the pustule secretion of a milk maid suffering from cowpox, whereupon the boy fell ill with a mild infection. Six weeks later, he inoculated the boy with smallpox secretion, and, as expected, the boy did not fall ill with the disease. Edward Jenner published his results two years later in a report that became a landmark paper in the *Annals of Medicine*. His statement ‘that the cowpox protects the human constitution from the infection of smallpox’ laid the foundation for modern vaccinology (Jenner). His method underwent medical and technological changes over the next 200 years, and eventually resulted in the eradication of smallpox in 1980 (Breman, Arita and WHO 1980).

The first compulsory vaccination requirements were passed in Italy in 1806, France in 1810, and in Sweden in 1816 (Salmon *et al*. 2006). In the UK, Parliamentary Acts of 1853 and 1867 made the vaccination of children against smallpox compulsory, and were backed by fines and even imprisonment. However, distrust of the medical profession and governments was a major factor fuelling resistance to vaccination, and in the late 19th century, thousands took to the streets in England demanding the repeal of the Vaccination Acts. Following mass protests in the 1880s,  arguing that individual rights were being sacrificed for the benefits of the population as a whole, a Vaccination Act of 1898 introduced an opt-out clause based on conscientious objection (El Amin *et al*. 2012).

The next advance in the development of vaccines, using the principle of attenuation, was invented by Louis Pasteur, nearly a century after Edward Jenner's experiments. In 1880, Pasteur succeeded in producing a vaccine against cholera in chickens caused by *Pasteurella multocida* (Pasteur 1880). Pasteur's pupil Émile Roux was able to prove the active principle of this immune defence by blood tests. Only one year later, an effective vaccine against anthrax was developed (Lombard, Pastoret and Moulin 2007). In 1884, Pasteur, for the first time, cured a patient infected with the pathogen causing rabies by means of a successful vaccination (Bardenave 2003). This contained material obtained from infected rabbit brain attenuated by drying, an unreliable process, and vaccines prepared in this way frequently caused serious side effects. Nevertheless, Pasteur's attenuation principle marked the beginning of the process by which laboratory science would transform vaccine development during the course of the subsequent century.

Between 1890 and 1950, bacterial vaccine development proliferated, based on the discovery that immunogenicity could be retained if bacteria were killed by heat or chemical treatment (vaccines against typhoid, plague, cholera and pertussis), or by administration of inactivated bacterial toxin (e.g. diphtheria, tetanus) (Behring and Kitasato 1890). In 1954, a further breakthrough was achieved with the discovery that cells could be cultured *in vitro* and used as substrates for viral growth. This discovery would spawn an entirely new generation of vaccines—of killed or attenuated viruses—including polio, measles, mumps, rubella, adenovirus and others (Enders, Weller and Robbins 1949; Salk 1955; Gotschlich, Liu and Artenstein 1969; WHO 2020a). The polio vaccines (Sabin, Hennessen and Winsser 1954) have eliminated polio from most of the world and reduced the number of reported cases from an estimated 350 000 cases in 1988 to 33 in 2018 (WHO 2020a).

This was the phenomenal 20th-century legacy to global public health and the 21st century started at a similar pace with the development of novel vaccines: an extended spectrum of polysaccharide conjugates against encapsulated bacteria, vaccines against rotavirus diarrhoea and vaccines against oncogenic papillomavirus promising the elimination of cervical cancer and other sexually transmitted tumours in both sexes (Cryz *et al*. 1987; Peltola 2000; Villa *et al*. 2006; Pallecaros and Vonau 2007; Turner *et al*. 2017). In parallel, ‘universal vaccination’ became increasingly global, thanks to unprecedented coordinated efforts by the WHO Expanded Programme on Immunization, national governments, the vaccine industry and philanthropic foundations to make vaccines accessible to all children, regardless of means.

## MODERN VACCINOLOGY

Vaccination has been a major advance for healthcare, allowing the eradication and reduction of various infectious diseases. While existing vaccines continue to be improved and distributed, major pathogens such as human immunodeficiency virus (HIV) or the causative agent of malaria represent significant research challenges that require the development of new vaccination strategies. An additional challenge—the focus of this paper—is developing vaccines in preparation for and during pandemics. Moreover, vaccine development is a field that requires constant adaptation in the face of a changing pattern of infectious diseases, hence a need for better education, communication, and engagement with health professionals and the public about this sensitive field. Here we identify key elements of apprehension.

### Access to vaccines and vaccination

In various countries, the vaccination coverage is reduced due to limited access to vaccines and vaccinations. This is relevant not only to low- and middle-income countries with weak healthcare infrastructures but also to high-income countries, which do not always take into consideration the daily life barriers of the population. Easy access to vaccination needs to be accomplished by barrier-free access.

### Demographic changes

In response to public health policies, life expectancy has doubled over the last century and the proportion of the elderly in the population continues to rise. Elderly people are more susceptible to infectious diseases and less responsive to vaccination, as is clearly shown by their responsiveness to the influenza vaccine (Franceschi *et al*. 2017; Demicheli *et al*. 2018; Levett-Jones 2020). This growing situation warrants the adaptation of vaccines to the senescing immune system, thereby introducing a certain degree of personalisation at the scale of an age-defined population. For example, the Sanofi Pasteur Fluzone High-Dose trivalent flu vaccine and the Seqirus Fluad quadrivalent flu vaccine are specifically recommended for the over 65 years old (JAMA, published online: 2 October 2020) (Monto *et al*. 2009).

### Microbes change and new ones emerge

This is dramatically illustrated by the current worldwide COVID-19 pandemic. Examples of emerging diseases over the last 20 years include West Nile fever, influenza, severe acute respiratory syndrome (SARS), Middle East respiratory syndrome coronavirus (MERS-CoV) and antimicrobial resistant pathogens such as drug-resistant tuberculosis, gonococcus, extraintestinal *Escherichia coli* and*Staphylococcus aereus* (Cupertino *et al*. 2020).

### Attitudes change

Opposition to vaccination is not new, but the reasons why and the ways in which sections of the populations have developed opposition to vaccines have changed. The beginning of the 21st century was marked by a growing defiance by a small but vocal proportion of the population against vaccination and a subsequent decrease of vaccine coverage in many countries, particularly in Europe and North America. This decrease brings with it the threat of a widespread return of infectious diseases (e.g. measles, whooping cough, diphtheria) whose causative agents are still circulating in the population. Vaccination is therefore as much about relationships and trust between society and health authorities as it is medicine and science. Those actively opposing vaccination, while a small minority, can have an amplified view, particularly through online and social media activities, which offer them a larger forum. Consequently, there is an urgent need to re-establish confidence in vaccines. The major issue is to convince young parents of the importance of vaccination, and, in this context, paediatricians and general practitioners have a key role to play. This requires strengthening the training of medical students and post-graduates in preventive medicine, risk communication and vaccinology.

### Climate and environment change

Climate change and the ongoing change of natural environments by humans driven by increased urbanisation, global food production and exploitation of natural resources threatens habitats and reduces biodiversity. These pressures are a major driver of new zoonotic pathogens that are able to cross from one species to another. For example, expansion of the geographic range of the arthropod vectors had a considerable effect on the spread of arboviruses (Pena-Garcia, McCracken and Christofferson 2017; Sukhralia *et al*. 2019).

### Human behaviour changes

The reduction in travel and shipment costs has resulted in a significant and unprecedented increase in global trade and travel. As clearly shown by the COVID-19 pandemic, international travel is a major source of the global spread of emerging infectious diseases, which have the potential to develop into epidemics and pandemics. The global challenge is to be able to develop vaccines on a time-scale that is compatible with the control of pandemic spread or subsequent re-emergences. The pandemic spread of HIV, and now COVID-19, illustrates the difficulty of controlling and eliminating an emerging pandemic without the aid of a vaccine. Similarly, the virulent Ebola epidemics in Western Africa have also illustrated that when the dynamics of the epidemics are particularly robust, controlling an emergent disease simply by classical technologies (isolation and attenuation of the pathogen) is not realistic. Here again, only scientific progress in immunology, microbiology and vaccinology, coupled with international cooperation, will help to reduce the time necessary to conceive, develop and validate a vaccine.

## VACCINES IN OUTBREAKS AND PREPARATION FOR PANDEMICS

Preparing vaccines for potential pandemic outbreaks is complicated and challenging. There are two possible scenarios—an outbreak by a known pathogen (Ebola, Zika) and a new zoonotic pathogen (H1N1, swine influenza) (Vincent *et al*. 2014) or an outbreak by a new virus or an outbreak by a previously unknown pathogen or a new variant of a known pathogen. In the case of known pathogens, it is conceivable to develop a protective vaccine, which will reduce the risk of an epidemic. However, the challenge here is often mainly financial—developing a vaccine involves significant costs that have to be offset against their benefits. It is estimated (Gouglas *et al*. 2018) that the total cost of developing a vaccine for an epidemic infectious disease is a minimum of around 3 billion US dollars (see below). But this investment may bring little or no benefit if a pandemic does not develop. For ‘new’ pathogens, such as COVID-19, the development may not be fast enough and—also here—there is a risk that the pandemic is a one-time event that does not return, similar to the case of the 2002–2004 SARS outbreak. The case for developing a vaccine protective against COVID-19 is unique because, in addition to the impact on health, the economic consequences are so severe. This has resulted in the need for considerable investment by governments and disease prevention charities. Together these funds have provided a financial blanket for vaccine developers.

**Figure 1.**
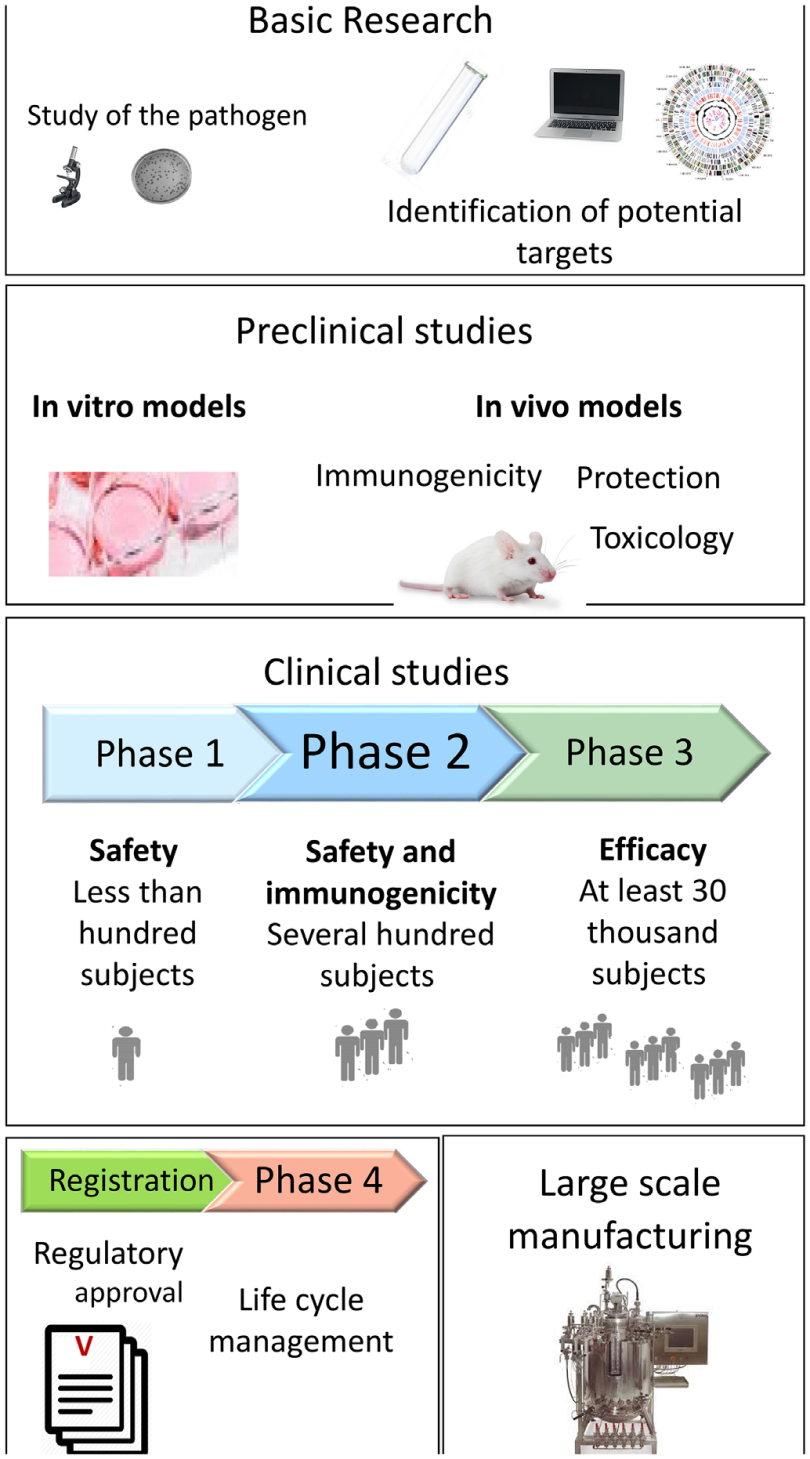
Stages in vaccine development.

As well as being enormously costly, the vaccine development process is generally lengthy and multidisciplinary. We outline the main steps needed to develop such a new vaccine and how this is funded—summarised in Fig. 1. Typically, research on a given microorganism starts in academia, examining questions such as disease pathology, transmissibility and characterisation of the agent in terms of taxonomy, structure, natural history, growth requirements, animal model development and deciphering immune responses that may lead to the identification of antigens and possibly correlates of protection. This research may include the identification of sub-structures, such as individual proteins or polysaccharides, particularly those at the infective agent's surface, that can be used in isolation or in combination as candidate immunogens. A key question is whether these antigens display variability among different isolates, both geographically and over time. Variability may imply the need for multivalency to be effective [e.g. vaccines against pneumococcus, meningitis, polio, human papillomavirus (HPV), etc.] or the need for seasonal changing of the vaccine (e.g. influenza). The vaccine development process then begins in earnest with the commitment to progress candidate immunogens to first-in-man studies, using clinical-grade materials prepared under conditions of strict regulatory compliance. The step towards clinical studies may be done in universities, often using the expertise and facilities of contract research organisations but is more typically carried out in industry. These clinical studies involve rigorous quality control and testing—for identity, purity, toxicity, consistency and quantitation. Candidate immunogens may be presented in various forms, ranging from live-attenuated whole organisms, killed or inactivated whole organisms, nucleic acid vaccines, vectored genes encoding the protein antigen of interest through to individual ‘sub-units’ of purified proteins or protein complexes or free or conjugated polysaccharides. Methods of delivery are typically intramuscular (IM) or subcutaneous injection but can be oral or nasal, as is the case with some live-attenuated vaccines (LAVs).

Development of clinical-grade material allows progression of the immunogen through the clinical development phases, which are typically Phase I for safety, Phase II for immunogenicity, and phase III for efficacy, large-scale safety and product consistency, preferably from intended final production facilities. Eventual approval and licensure require ongoing pharmacovigilance and possibly Phase IV studies for extending the use of the vaccine. The wide range of product types (killed, LAV, sub-unit, nucleic acid, etc.) means that generally each new vaccine requires the development of its own specific technology and protocols, for production, downstream processing (purification and optimisation), quality control and even filling and packaging. Typically, each vaccine is produced in a bespoke and dedicated facility, meaning that bioproduction in multiuse facilities is technically difficult and uncommon. Although there are concerted efforts nowadays to develop platform technologies that can be used for the development and manufacture of a range of vaccines, and for attempts to shorten the costs and timelines involved, the research and development (R&D) process outlined above typically takes 10–15 years and the costs have been variously estimated to be around $800 million but can be significantly higher, driven by the scale and complexity of Phase III studies. In addition, the construction of large-scale manufacturing facilities requires further investment, which may be in the range of $500 million and the work typically takes four or more years. This latter investment is often required to be ‘at risk’, that is to say, it is needed before proof of product and efficacy has been established, e.g. during Phase II, in order to be ready to launch the product and meet demand in major markets and affected areas immediately after licensure.

The cost, long-term commitment, know-how, supply chain management, etc., make the above scheme in its entirety manageable only by major pharmaceutical companies and only for vaccines that are required across large areas of the world, including wealthy countries with developed healthcare systems, and whose uptake will be universal or at least very widespread. It is normally not possible for companies to make a commitment to spend in excess of $1 billion without an expectation of a ‘return on investment’ through sales of vaccine in the years that follow. Spending on vaccine projects that will not even return the cost of development is a recipe for the demise of the industry and the potential disruption to the supply of existing vaccines. Also, it is self-evident that the above scheme does not work well economically for vaccines that are required for very restricted regions, for vaccines needed only for short periods to address outbreaks or for vaccines needed only in lower income countries that cannot afford to pay prices that would ensure a return on investment. Thus, the business case against the development of vaccines for diseases such as Ebola, Lassa, Hendra, Zika and some others on the WHO priority list is not positive.

Fortunately, there are methods and means to enable and incentivise vaccine development when the business case is lacking. These are often referred to as ‘push-and-pull’ measures. ‘Push’ is where governments, charities, etc., fund early-stage research in an attempt to secure a proof of concept and thus de-risk the scientific aspects of the project. ‘Pull’ is structured so that sales of certain volumes of vaccine at a pre-arranged price can be agreed between the health authorities and the manufacturer ahead of the launch so that revenues in the early years are guaranteed.

Historically, the most spectacular grassroots, non-industrial funding of a vaccine project was the March of Dimes, organised by the National Foundation for Infantile Paralysis (NFIP) founded by Franklin D. Roosevelt. The US population gave generously to this mission to provide grants to researchers studying the poliovirus, culminating in the development of the Salk inactivated polio vaccine and, subsequently, the Sabin live-attenuated orally administered polio vaccine. More recently, the Bill and Melinda Gates Foundation announced as its goal ‘to identify, support, and shape scientific research that can have the most impact and to accelerate the translation of scientific discoveries into solutions that improve people's health and save lives’. This announcement has provided a serious infusion of funds into R&D of vaccines targeted at the developing world. Other charities such as the Wellcome Trust, The Hillman Foundation as well as government-supported institutes have contributed significantly to the funding of the fundamental science of vaccinology.

Thus far, however, it has been difficult to amass the funding, from outside the private sector, to complete a vaccine development process through Phase III to licensure. A recent notable exception to this and the heartening success is the development and production of an Ebola vaccine for use in outbreak control in Western Africa. This was achieved with significant international support and with both philanthropy and know-how support from the Merck organisation in collaboration with NewLink Genetics. The Ebola outbreak, however, did illustrate that the world is ill-equipped to deal with outbreaks that may initially threaten restricted geographical regions but then may broaden to much wider geographical regions. These lessons learnt from the Ebola outbreak were one of the drivers for the creation in 2017 of the Coalition for Epidemic Preparedness Innovations (CEPI) to develop vaccines against emerging infectious diseases. CEPI is funded by donations from public, private, philanthropic and civil society organisations and is supported by the main vaccine manufacturers. Originally focused on the WHO's ‘blueprint priority diseases’, CEPI has emerged in 2020 as key player in the race to develop a vaccine against COVID-19.

## SOCIAL AND BEHAVIOURAL ASPECTS OF VACCINATION

To determine possible public reactions to a newly developed SARS-CoV-2 vaccine, it is helpful to look at vaccines that were rolled out in response to other disease outbreaks. Interestingly, a variety of different reactions can be found. In the 1950s, there was a high demand for the polio vaccine because the risk was very present. On the other hand, during the 2009 H1N1 ‘swine flu’ pandemic, the concerns over a new vaccine being rushed or inadequately tested led to low uptakes in some countries.

Those who are critical of or who are opposed to vaccination might now be expected to be quiet in the face of a real-life reminder of a time before vaccines controlled many debilitating fatal diseases. However, as the prospect of new vaccines enters development with a number of candidates in Stage II and even Stage III trials, the promise of a vaccine against COVID-19 becomes more concrete. At the same time, the position of those who oppose vaccination becomes more apparent. Some celebrities and high-profile personalities have already spoken out against a possible vaccine. Even more concerning, some of those who protested against lockdown measures in the USA and promoted fake news stories about the safety of new vaccines have been shown to be members of vaccine-critical groups (Hotez 2020a, b).

It appears that the heightened stakes of an infectious disease threat can push certain factions of the population into a more entrenched, non-science-based position, which includes a stronger and more vocal opposition to vaccination. There are a number of reasons why vaccine critiques are especially worried that their values and beliefs will not be upheld during a global health crisis. These worries extend from the suspicion and mistrust of governments, pharmaceutical companies and international organisations across many areas, such as encroachment into private lives (through surveillance and possible enforcement of vaccines). Moreover, the steady increase in misinformation and disinformation, and ‘influencers’ offering alternative ‘natural cures’ for COVID-19 that are either ineffective or even harmful pose additional risks (Pennycook 2020). Scepticism** about the motivations of the developers of new vaccines and questions about safety and usefulness could potentially derail a vaccination campaign as has happened previously. For example, in 2017, a rumour about the vaccination making children infertile halted a government measles and rubella vaccination campaign in five Indian states (Palanisamy, Gopichandran and Kosalram 2018).

Even though a vaccine for COVID-19 will go through rigorous safety and effectiveness tests, a level of apprehension is likely to exist among the public and in the media about the integrity of vaccine trials and possible adverse side effects. It is, therefore, most important that Phase IV post-marketing studies are done transparently by governments, to inform the public about the efficacy in particular risk or target groups, the side effects and the risks associated with non-vaccination. Health authorities and governments will also need to react quickly to false or misleading information in cooperation with social media and messaging companies. These companies are aware of their role against fake news, as shown by the steps taken by the private messaging service WhatsApp, which is owned by Facebook. WhatsApp recently made changes to limit ‘frequent forwards’, which restricted the number of times that a message can be forwarded to five (Hern 2020). Further vigilance and interventions will still be needed to address opposition to vaccination and fake news.

## NEW TECHNOLOGIES AND HOW THEY CAN PROVIDE A SOLUTION TO NEW THREATS TO HUMAN HEALTH

Increasing knowledge of chemistry, immunology, microbiology, molecular biology and structural biology and the access to new technologies have completely changed the vaccine development landscape, providing the tools for the development of new, more effective and safer vaccines.

The discovery that chemical conjugation of a polysaccharide makes the sugar immunogenic and is able to induce a strong T-cell-dependent response, helping B-cells to produce high-avidity antibodies and immune memory (Costantino, Rappuoli and Berti 2011), was a significant breakthrough. It facilitated the development of conjugated vaccines that are efficacious for the prevention of diseases, such as *Haemophilus influenzae* type B (Peltola *et al*. 1977), four meningococcal serogroups (A, C, W and Y) and up to 21 *Streptococcus pneumoniae* serotypes, with many more in pre-clinical and clinical development. The application of genetic engineering and molecular biology techniques means that antigens can be expressed in heterologous microorganisms, and this has facilitated the rational design of immunogenic and safe antigens. The first vaccine to be designed using genetic engineering technologies was the hepatitis B vaccine, in which the gene encoding the main antigen, the viral capsid's ‘S’ antigen, was cloned and expressed in a heterologous expression system. The antigen was expressed in yeast at a very high yield and self-assembled into virion-like structure resembling the viral coat. This antigen was highly immunogenic and much safer compared to the previous hepatitis B vaccine, in which viral particles devoid of their nucleic acid were purified from the blood of infected individuals. The recombinant hepatitis B vaccine is widely used for immunisation starting from 2 months of age, and in many countries, it is given within 24 hours from birth. The heterologous expression system of viral antigens is being applied to many other viruses, such as the HPV and the herpes zoster virus. Three HPV vaccines have been registered so far, based on two, four or nine serotypes expressed as virus-like particles in insects or in yeast.

Recombinant DNA technologies have been also successful in the case of bacterial vaccine developments. The first example was the design of a recombinant non-toxic and immunogenic pertussis toxin, the main virulence factor of *Bordetella pertussis* (Marsili *et al*. 1992). In this case, a mutant pertussis toxin is produced by an engineered *B. pertussis* strain, in which the two codons identified by sequence alignment as being essential for the adenosine diphosphate (ADP)-ribosylation activity have been mutagenised. The resulting inactive gene was used to replace the wild-type gene on the *B. pertussis* chromosome, by allelic recombination. The PT9K/129G mutant toxin, purified from the culture supernatant of the recombinant strain, has been shown to be safe, able to induce neutralising antibodies and efficacious in clinical trials. The basic principles for the design of non-toxic forms of bacterial toxin, through site-directed mutagenesis or expression of only immunogenic domains, are now routinely applied to the development of other bacterial toxins as antigens or as carrier proteins for polysaccharide vaccines.

Genetic engineering technologies are now routinely used to generate rationally designed attenuated bacterial strains. This approach involves the identification of genes encoding virulence functions and their inactivation to generate non-toxic but immunogenic strains. The main challenge resides in the ability to identify bacterial functions, which do not impact on the ability of the strain to colonise and replicate in the host to induce an effective immune response while impacting on the strain's ability to cause disease. Such approaches have been extensively applied, mainly to enterotoxigenic pathogens such as *Salmonella enterica* subsp. *enterica* Serovar Typhi, *Vibrio cholera* and *Shigella* strains (Barry and Levine 2019). Although very promising, no vaccines based on this technology have so far been licensed, suggesting that the right balance between reduced virulence and immunogenicity continues to be a challenge.

Engineered *E. coli* strains have been recently used to produce bio-conjugated polysaccharide vaccine antigens. In this case, both the polysaccharide and the carrier protein are synthetised in the *E. coli* bacteria and conjugated by the PglB oligosaccharyltransferase enzyme (Wacker *et al*. 2006; Huttner *et al*. 2017). This methodology avoids the purification of the separate antigens (the polysaccharide and the carrier protein) and the need for their subsequent chemical conjugation. Bioconjugates for *Shigella flexneri* and extraintestinal *E. coli* have been tested in a Phase I clinical trial with promising results (Hatz *et al*. 2015; Huttner *et al*. 2017). Additional bio-conjugate vaccines are in pre-clinical studies.

Gram-negative bacterial strains can also be engineered to produce high yields of outer membrane vesicles (OMVs), known to be enriched with outer membrane proteins that represent ideal vaccine antigens. Strains are engineered through the introduction of chromosomal mutations generating an ‘overblebbing’ phenotype and a less reactogenic lipopolysaccharide (LPS). OMVs derived from these recombinant strains, also named GMMAs (generalised module membrane antigens), can be further engineered to over-express heterologous antigens and to form the basis of multivalent vaccines (Berti and Micoli 2020). The immunogenicity of *Shigella sonnei* GMMA has been shown in a Phase I clinical trial in humans (Obiero *et al*. 2017).

The genomic era has changed the vaccine landscape even more. Starting from the first genome sequence of a bacterial pathogen, *Haemophilus influenzae* type B, the number of sequenced genomes available in the European Bioinformatics Institute (EBI) and National Center for Biotechnology Information (NCBI) databases is of the order of a hundred thousand. The genome revolution has been possible because of the rapid whole genome sequencing technologies that allow an entire genome to be sequenced within minutes and at very low cost. Therefore, whole genome sequencing is easier than the ‘one-by-one’ sequencing of a few genes of interest and provides a comprehensive picture of the sequences responsible for the epidemiological variability and virulence features of a given microorganism. Moreover, the genome sequence provides access to the antigens that are potentially expressed at all stages of its virulence cycle. Access to this comprehensive set of information has facilitated the introduction of a breakthrough technology referred to as ‘reverse vaccinology’. The principle is based on the possibility of screening a genome *in silico*, using a wide number of bioinformatic tools that can drive the identification of surface-exposed antigens, a family of proteins that have a high probability to be efficient targets for antibody recognition. The first success of the ‘reverse-vaccinology’ approach has been the development of the multivalent meningococcal vaccine, named 4CMenB.

Bioinformatic screening of the meningococcal B genome strain MC58 resulted in the identification of 600 potential surface-exposed or secreted antigens. The genes encoding these potential antigens were amplified and cloned in *E. coli* on an expression vector. A total of 350 antigens were successfully purified, used to immunise mice and antisera screened in a wide range of *in vitro* assays (Pizza *et al*. 2000). Among the 96 new surface antigens, 3 induced antibodies with complement-mediated bactericidal activity against a variety of meningococcal strains, a property known to correlate with protection in humans. The three antigens, combined with the OMVs of an outbreak strain (the New Zealand strain), are now the basis of a meningococcal vaccine, which is licensed for vaccination starting from 2 months of age in more than 40 countries and for use in adolescents in the USA (Masignani, Pizza and Moxon 2019). The potential of the ‘reverse-vaccinology’ approach to identify novel antigens as potential vaccine candidates has subsequently been demonstrated for a wide range of bacterial pathogens such as Group A and Group B *Streptococcus*, *Staphylococcus aureus* and extraintestinal *E. coli*. Many of the newly discovered antigens are under analysis for their immunological properties in pre-clinical studies and are expected to be tested in clinical studies in the near future (Delany, Rappuoli and Seib 2013).

The revolution in the vaccine field brought about by the genomic era is based around the ability to access genomic data through an open-source network. For example, a novel virus can be sequenced overnight, and its genome sequence made available immediately worldwide. Candidate vaccine proteins can then be identified and DNA synthesis technology used to generate the required viral genes, or the whole virus itself, for subsequent expression in prokaryote or eukaryote production systems. Once the genome sequence is made publicly available, genes encoding for the capsid antigens can be synthetised and cloned in non-pathogenic viruses attenuated in their ability to replicate in host cells, or in non-replicating viruses. Viral vectors widely used are poxvirus, alphavirus and adenovirus. More recently, vesicular stomatitis and measles virus were also considered as promising viral vectors (Lauer, Borrow and Blanchard 2017).

The case of influenza strain H7N9 is a good example of the power of synthetic biology to accelerate vaccine development. The genome sequence of H7N9 was made available online by a Chinese group in March 2013. Researchers at Novartis Vaccines, in collaboration with the Institute of Synthetic Genomics, used the sequence data to synthesise the viral genes and insert them into a viral vector that was then used to infect eukaryotic cells to produce a recombinant virus. As a result, in just a few days, the vaccine seeds were ready to be used for manufacturing (Dormitzer *et al*. 2013). The recombinant viruses could then be used to infect the target cells, which produce the heterologous viral antigens and induce strong B- and T-cell responses (Pinschewer 2017).

A recent example of the use of viral vectors relates to the development of the Ebola vaccine (Tully *et al*. 2015). The vesicular stomatitis virus (VSV)–Ebola virus (EBOV) vaccine is based on the Ebola glycoprotein cloned into a VSV and was approved by the European Medicines Agency (EMA) and US Food and Drug Administration (FDA) in 2019. The cAD3-EBO Z vaccine is based on an attenuated version of a chimpanzee adenovirus, genetically engineered to be unable to replicate in humans, and to express on its surface the Ebola glycoprotein. This vaccine was tested in a Phase III clinical trial. The Ad26.ZEBOV/MVA-BN-Filo vaccine, in which the human adenovirus serotype 26 (Ad26) is expressing the Ebola glycoprotein and the MVA-BN (Modified Vaccinia Virus Ankara—Bavarian Nordic) vaccine vector, was tested in a prime-boost immunisation regimen. Marketing authorisation for this vaccine was submitted in 2019.

Synthetic biology is also the basis of another major technological innovation in which antigen-encoding messenger RNA (mRNA) acts as a vaccine platform. mRNA is non-infectious, does not integrate into the chromosome, does not self-replicate and is naturally degraded by the host cells (Pardi, Hogan and Weissman 2020). Efficient delivery of mRNA can be achieved through formulation with carrier molecules such as liposomes, cationic polymers or nanoemulsion, which increase uptake and antigen expression. Self-amplifying mRNA (SAM) vaccines are based on an alphavirus genome, in which the genes encoding for the RNA replication system are preserved, while the genes encoding the structural proteins are replaced with the antigen of interest. The mRNA technology has been applied to a variety of infectious viral and bacterial pathogens; the field is evolving very rapidly, and many pre-clinical and clinical studies are ongoing. It has been shown that mRNA vaccines induce potent CD4+ and CD8+ T-cell responses and, unlike the DNA-based vaccines, are able to generate potent neutralising antibody responses in animals after only one or two doses. The main advantage of mRNA vaccines is that they are synthesised *in vitro* and therefore do not require growth in cell culture and can be produced rapidly at a relatively low cost. However, recently published results from two clinical trials on influenza and rabies mRNA vaccines showed a lower response compared with what was expected from the pre-clinical data (Pardi, Hogan and Weissman 2020).

Access to multiple genome sequences also provides data on the strain-to-strain sequence variations of specific antigens, allowing the design of recombinant antigens predicted to provide cross-protection against genetically diverse strains. Data on strain-to-strain sequence variability, combined with the knowledge of the three-dimensional structure and epitope mapping, could guide the design of more stable, more immunogenic and cross-reactive antigens. This multifactorial approach is the basis of the ‘structural-vaccinology’ era, also referred to as ‘reverse vaccinology 2.0’ (Rappuoli *et al*. 2016). The success of such an approach was elegantly shown by the RSV (respiratory syncytial virus) vaccine, based on a stable and strongly immunogenic ‘pre-F’ antigen (McLellan *et al*. 2013). The RSV F antigen consists of two forms: pre- and post-fusion. The pre-fusion protein is the antigenic form that mediates the fusion between the viral capsid and the host cell membrane. Following the fusion, the F capsid antigen undergoes a conformational change, assuming an open structure (post-fusion). Antibodies with neutralising activity recognise the pre-fusion form of the protein and block its interaction with the host cell receptor. When expressed as a recombinant protein, the pre-fusion protein has an unstable conformation, which opens up, shifting its structure to the post-fusion conformation. The introduction of two cysteine residues in key positions allowed the formation of a disulphide bond, which locks the F protein in its pre-fusion, and therefore immunogenic, structure. This stabilised F protein induces neutralising antibodies in humans of one order of magnitude higher than the non-stabilised form. Clinical trials are ongoing, and this approach is expected to deliver an effective RSV vaccine in the near future. Additional RSV vaccine approaches include adjuvanted formulations and mRNA (Higgins, Trujillo and Keech 2016).

One of the principles of the reverse vaccinology 2.0 is based on the generation of human monoclonal antibodies from convalescent or vaccinated subjects for the identification of new protective antigens or for the mapping of protective epitopes. This kind of approach has been successful in the identification of the most protective cytomegalovirus antigen based on a pentameric capsid antigen, which is under evaluation in pre-clinical studies (Macagno *et al*. 2010).

The same approach has been used in the case of influenza for the identification of a universal flu vaccine effective against all subtypes of this virus (Krammer and Palese 2015). A cross-reactive monoclonal antibody (mAb) was identified as being able to recognise 16 different haemagglutinin (HA) subtypes. The mAb mapped to a conserved epitope located on the stem of the HA protein, the region that varies less frequently compared with the head region of this protein. An influenza vaccine based on the HA stem antigen was able to induce broadly neutralising antibodies in an animal model (Nachbagauer *et al*. 2018). Moreover, three monoclonal antibodies, isolated from a patient infected with the influenza A H3N2 virus, were able to bind to the neuraminidase active site, neutralising the virus across multiple strains.

Finally, the advances in the field of immunology have allowed the discovery of novel adjuvants, molecules that, when added to vaccine antigens, are able to enhance its immunogenicity. The need for adjuvants is particularly important for purified recombinant antigens, which induce a lower immunogenicity compared to live-attenuated or inactivated whole-cell vaccines, which contain many immune-stimulatory components.

MF59, a squalene oil-in-water emulsion, is the adjuvant used in the seasonal influenza vaccine, licensed for use in individuals of 65 years and over. This adjuvant can induce recruitment of inflammatory cells at the injection site, resulting in increased antigen uptake and stimulation of adaptive immunity. MF59-adjuvanted influenza vaccine showed higher efficacy than the non-adjuvanted vaccine in preventing laboratory-confirmed influenza and hospitalisations (Domnich *et al*. 2017). Adjuvant systems are combinations of immunostimulatory molecules, which have been recently licensed for adjuvanted vaccines such as the papillomavirus vaccine containing AS04, an aluminium salt formulated with 3-odesacyl-4′-monophosphoryl lipid A (MPL), a detoxified form of LPS extracted from *Salmonella minnesota*; the influenza vaccine containing AS03, an oil-in-water emulsion, containing squalene and α-tocopherol (vitamin E) as an immunostimulant; and the malaria and the herpes zoster vaccines containing AS01, composed of two immunostimulatory molecules, MPL and saponin QS-2, a triterpene glycoside purified from the bark extract of *Quillaja saponaria* Molina (fraction 21) (Del Giudice, Rappuoli and Didierlaurent 2018). CpG ODN, a soluble oligonucleotide TLR9 agonist and RC-529, a synthetic glycolipid TLR4 agonist, are adjuvants for two commercial hepatitis B vaccines, immune stimulating complexes (ISCOMs) and Matrix M, based on phospholipid and cholesterol nanocomplexes and saponins, are the adjuvants for influenza vaccines (Apostolico Jde *et al*. 2016). These adjuvants contributed to enhancing the immune response significantly compared to non-adjuvanted vaccines, in terms of antibody and cell-mediated immunity, and efficacy and effectiveness.

## CORONAVIRUS VACCINES

**Figure 2.**
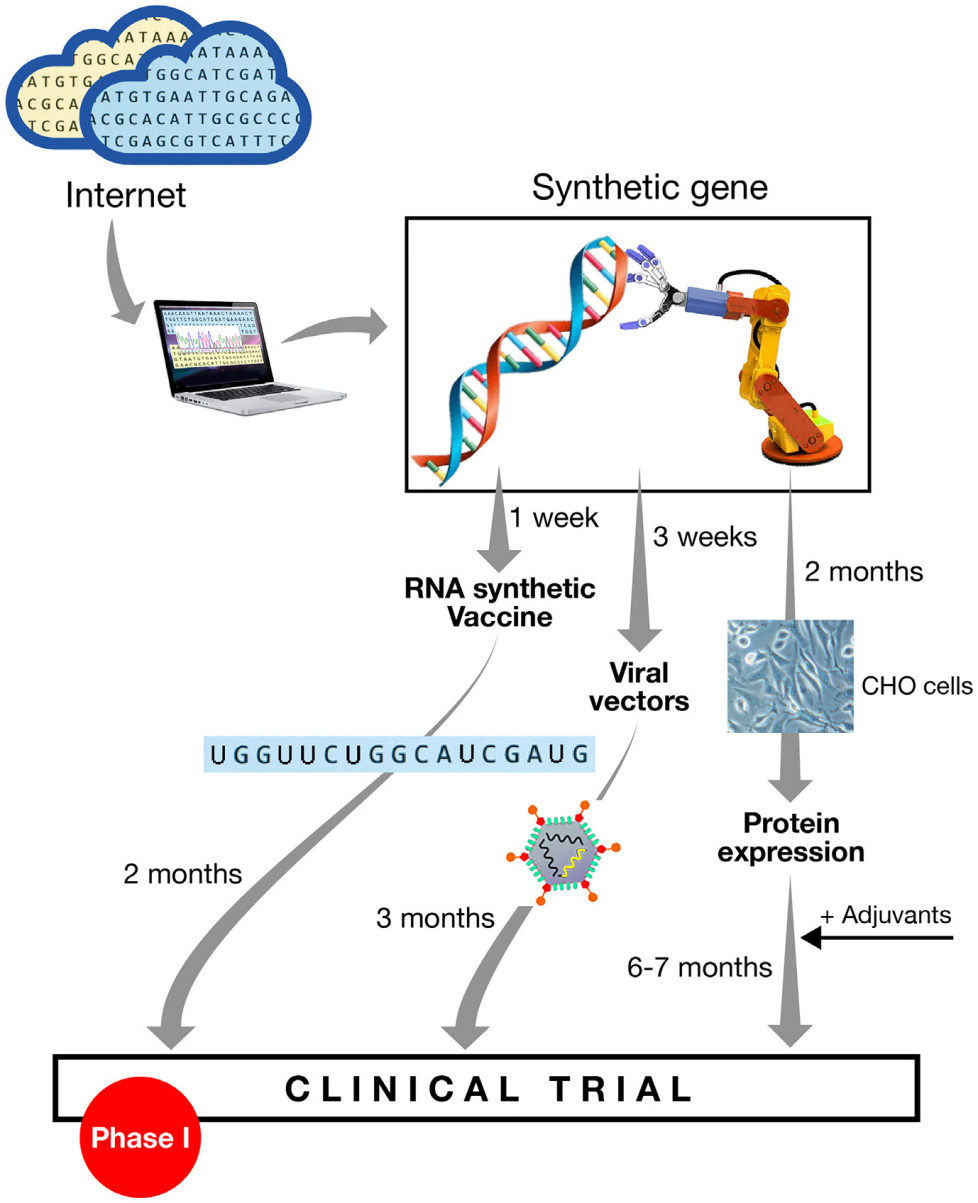
Development of vaccine in times of COVID-19.

A safe and effective vaccine that is able to prevent disease, infection and spread of SARS-CoV-2 is urgently needed. The current COVID-19 pandemic represents a clear example on how the new technologies can drive vaccine design quickly and effectively (Fig. 2). Immediately after SARS-CoV-2 was identified as the infectious agent, and the genomic sequence made available, the search for vaccines and therapeutics was initiated. Academic research groups, small biotech companies and large pharmaceutical companies have started to share data and to cooperate. As of November 2020, more than 200 candidate vaccines have been proposed and 47 are being tested in clinical studies (Table 1, https://www.who.int/publications/m/item/draft-landscape-of-covid-19-candidate-vaccines). The main approaches include killed inactivated virus, viral vectors, virus-like particles, DNA, RNA and sub-unit vaccines. The mRNA vaccine developed by BioNTech/Pfeizer was shown to be 90% effective in preventing COVID-19 infection in the first interim analysis of a Phase III study conducted on 43 538 subjects. Results of the Phase III studies with the mRNA Moderna vaccine showed it to be 94.5% effective. Additional candidate vaccines that are expected to enter a clinical trial in 2020 are based on the same technologies described above and include alternative delivery routes, such as oral or intranasal.

**Table 1.** COVID-19 vaccines in clinical studies.

Manufacturer	Vaccine type	Antigen	Clinical stage
Sinovac	Inactivated	Killed virus	Phase III
Sinopharm/Wuhan Institute of Biological Products	Inactivated	Killed virus	Phase III
Sinopharm/Bejing Institute of Biological Products	Inactivated	Killed virus	Phase III
University of Oxford/Astrazeneca	Non-replicating adeno vector	ChAdOx1–5	Phase III
CanSino Biolocal, Inc./Bejing Institute of Biotechnology	Non-replicating adeno vector	Adeno virus Type 5 Vector	Phase III
Gamaleya Research Institute	Non-replicating adeno vector	rAd26-S + rAd5-S	Phase III
Jannsen Pharmaceutical Companies	Non-replicating adeno vector	Ad26COVS1	Phase III
Pfeizer/BioNTech	RNA	LNP encapsulated mRNAs	Phase III
			**Interim analysis 95% efficacy**
Moderna/NIAID	RNA	LNP encapsulated mRNA—S	Phase III
			**Interim analysis 94.5% efficacy**
Curevac	RNA	mRNA	Phase III
Novavax	Protein sub-unit	S nanoparticle Matrix M adjuvant	Phase III
Sanofi Pasteur/GSK	Protein sub-unit	S + AS03 adjuvant	Phase III
Medicago/GSK/Dynavax	Virus-like particles	Plant-derived VLPs + adjuvant	Phase III
Clover Biopharmaceutical/GSK/Dynavax	Protein sub-unit	Trimeric S + adjuvant	Phase II/III
Israel Institute for Biological Research/Weizmann Institute of Science	Replicating viral vector	VSV-S	Phase II
Arcutus/Duke	RNA	mRNA	Phase II
Inovio Pharmaceuticals/International Vaccine Institute	DNA	DNA with electroporation	Phase II
Osaka University/AnGes/Takara Bio	DNA	DNA + adjuvant	Phase II
Cadila Healthcare Limited	DNA	DNA	Phase II
Genexine Consortium	DNA	DNA	Phase II
Anhui Zhifei Longcom Biopharmaceutical/Institute of Microbiology, Chinese Academy of Science	Protein sub-unit	RBD-dimer + adjuvant	Phase II
Bharat Biotech	Inactivated	Killed virus	Phase I/II
Institute of Medical Biology, Chinese Academy of Medical Science	Inactivated	Killed virus	Phase I/II
Research Institute for Biological Safety, Republic of Kazakhistan	Inactivated	Killed virus	Phase I/II
Beijing Minhai Biotechnology	Inactivated	Killed virus	Phase I/II
Kentucky Bioprocessing	Protein sub-unit	RBD	Phase I/II
SpyBiotech/Serum Institute of India	Virus-like particles	RBD-HBsAg VLPs	Phase I/II
ImmunityBio, Inc. & Nantkwest, Inc.	Non-replicating Adeno vector	hAd5 S + second-generation hAd5 S + nucleocapsid	Phase I
ReiThera/LEUKOCARE/Univercells	Non-replicating adeno vector	Simian adenovirus-S	Phase I
Cansino Biologicals, Inc./Institute of Biotechnology, Academy of Military Medical Sciences	Non-replicating adeno vector	Ad5-nCov (IM and mucosal)	Phase I
Vaxart	Non-replicating adeno vector	Ad5-adjuvanted oral	Phase I
Ludwig Maximilians University of Munich	Non-replicating vector	MVA-SARS-2-S	Phase I
Merck/IAVI	Replicating viral vector	Replication-competent VSV delivering spike S	Phase I
Institute Pasteur/Themis/University of Pittsburgh/Merck	Replicating viral vector	Measles-vector based	Phase I
Beijing Wantal Biological Pharmacy/Xiamen Univeristy	Replicating viral vector	Flu-based RBD intranasal	Phase I
Imperial College London	RNA	LNP-nCovsaRNA	Phase I
People Liberation Army Academy of Military Science/Walvax Biotech	RNA	mRNA	Phase I
Symvivo	DNA	bacTRL-S	Phase I
		Oral	
Vaxine Pty Ltd/Medytox	Protein sub-unit	S + Advax adjuvant	Phase I
			
University of Queensland/CSL/Seqirus	Protein sub-unit	Molecular clamp stabilised spike + MF59 adjuvant	Phase I
Medigen Vaccines Biologics Corporation/NIAID/Dynavax	Protein sub-unit	S-2P + CPG adjuvant	Phase I
Instituto Finlay de Vacunas, Cuba	Protein sub-unit	RBD conjugated to tetanus toxoid	Phase I
Instituto Finlay de Vacunas, Cuba	Protein sub-unit	RBD + adjuvant	Phase I
FBRI SRC vb Vector, Koltsovo	Protein sub-unit	Peptide	Phase I
West China Hospital, Sichuan University	Protein sub-unit	RBD	Phase I
University Hospital Tubingen	Protein sub-unit	HLA-DR peptides subcoutaneously	Phase I
Covaxx/United Biomedical, Inc., Asia	Protein sub-unit	Multiepitope-based RBD	Phase I

S: spike protein, RBD: receptor binding domain; HBsAg: hepatitis B antigen; VSV: vesicular stomatitis virus; LNPs: lipid nanoparticles; bacTRL: bacterial vector in probiotic bacteria.

Although it is not known how many of these vaccines will show efficacy and persistence in humans, the major challenge will be to generate an extremely high number of doses rapidly in order to vaccinate the global population, at least those that are defined as populations at risk. In practice, it is likely to be necessary to license more than a single vaccine. One possibility for improvement is the use of an adjuvant for the sub-unit-based vaccines. The addition of an adjuvant such as Matrix M, AS03, MF59 or CPG could increase the immune response at a lower antigen dose, resulting in the availability of a much higher number of vaccine doses. Adjuvants are easy to manufacture and are expected to provide a solution for a response to a rapidly evolving global emergency.

## CONCLUSIONS

There is no doubt that vaccines have made an enormous contribution to the health and well-being of humanity and have shaped civilisation profoundly over the last century. However, challenges remain and the COVID-19 pandemic has shown that we cannot be complacent in the face of infectious disease and the ongoing need for effective vaccines against aggressive pathogens. For much of the later half of the 20th century, and up to December 2019, most populations, especially in high-income countries, were not seriously and universally threatened by infectious disease. This situation saw a dramatic change in 2020. The impact of COVID-19 on public health, as well as its economic and socio-political impact, has already changed the world and reminded us of the dangers we face when a deadly disease spreads and there is no effective intervention. The voice of anti-vaccine activists can still be heard on social media, but for the many it is a reminder of the value of vaccines and vaccination to our way of life.

The race for a COVID-19 vaccine is now at full speed with, at the time of writing, half a dozen or so candidates entering the later stages of clinical trials. Several of these, and many more in the pre-clinical pipeline, are harnessing new technologies, for example, those based on vector platforms recently developed to address SARS-1, MERS, Ebola and Zika outbreaks, or on recombinant proteins or the use of RNA or DNA. Others use more traditional approaches, relying on precedence to reduce the risk of failure. The scientific community has responded in earnest to the challenge and the pace of development is impressive. Guidance from regulators is needed to reduce the risk of adverse reactions and to ensure these efforts are successful. The design and development of an effective vaccine against SARS-CoV-2 is a challenge, but one that is based on a sound knowledge of the structure, function and immunobiology of related pathogens and a broad collective experience of vaccine development. Vaccination schedules of each and every country have also to be taken into consideration as both demographics and available vaccines will vary between countries. The global health community can be optimistic that at least one and probably several of the approaches will provide an effective vaccine, although the dangers of antibody-dependent enhancement and/or vaccine-enhanced respiratory disease cannot be dismissed. The development of a successful vaccine must be followed by a successful programme of manufacturing, distribution and vaccination. This will require an unprecedented scale-up of the successful technologies to produce the billions of doses of vaccine required, and their equitable and timely distribution throughout the world. Most of the technologies leading the race have never been scaled up to this extent and so rapidly, and it is uncertain whether they can deliver the quantities so desperately needed. As and when the supplies become available, the rollout and distribution of vaccine will require careful management and international support and good will based on effective and cooperative global politics. Apart from all these logistical issues, equity of access to vaccine has to be a high priority.

Yet, this situation raises additional questions: Who will own the vaccine? How to get it to those who need most? How can trials be ethically fast-tracked? There is no doubt that vaccine safety is as important as vaccine efficacy, urging the need for intensive safety studies both in the development phase and post-marketing, and then in larger population-based settings. Furthermore, while there is a large amount of focus on a possible COVID-19 vaccine, it is also important not to neglect routine immunisation programmes that protect people against vaccine-preventable diseases. There are additional challenges in delivering routine vaccination when health resources are directed elsewhere, as well as the difficulty or reluctance to visit doctors or places where vaccination takes place (such as schools). Utilising the vaccines that are already available is crucial in order to avoid other disease outbreaks on top of COVID-19. Moreover, when we eventually arrive in the post-COVID-19 era, world leaders must resolve to never allow this happen again by ensuring significantly strengthened R&D support for vaccine development and other infectious disease interventions and, in particular, generic effective future pandemic preparedness, as this is the fundamental basis of a strong public and global health architecture.
